# Use of High-Dose Chemotherapy in Front-Line Therapy of Infants Aged Less Than 12 Months Treated for Aggressive Brain Tumors

**DOI:** 10.3389/fped.2020.00135

**Published:** 2020-04-09

**Authors:** Milena Guidi, Laura Giunti, Anna Maria Buccoliero, Mariarita Santi, Barbara Spacca, Salvatore De Masi, Lorenzo Genitori, Iacopo Sardi

**Affiliations:** ^1^Neuro-Oncology Unit, Department of Pediatric Oncology, Meyer Children's Hospital, Florence, Italy; ^2^Medical Genetics Unit, Meyer Children's Hospital, Florence, Italy; ^3^Pathology Unit, Meyer Children's Hospital, Florence, Italy; ^4^Department of Pathology, Children's Hospital of Philadelphia, Philadelphia, PA, United States; ^5^Neurosurgery Unit, Meyer Children's Hospital, Florence, Italy; ^6^Clinical Trial Office, Meyer Children's Hospital, Florence, Italy

**Keywords:** congenital tumor, chemotherapy, glioblastoma, brain tumor, newborn

## Abstract

**Introduction:** Malignant brain tumors in infants less than 12 months of age are extremely rare, and they have poor prognosis. We evaluated genetic characteristics and response rates of infants with congenital brain tumors subjected to high-dose chemotherapy and autologous stem cell transplant after gross total tumor resection.

**Materials and Methods:** In total, 10 infants, aged less than 12 months, were enrolled in this study. The median age was 56 days (range: 1–279 days). Pathological examination demonstrated the following: four anaplastic astrocytomas, two glioblastomas, two central nervous system (CNS) embryonal tumors, not otherwise specified (NOS), and two atypical teratoid/rhabdoid tumors.

**Results:** All patients were exposed to induction chemotherapy regimen, two high-dose chemotherapy courses, and autologous stem cell transplant after maximal surgery. At 1–3–5 years, the global overall survival (OS) was 90, 70, and 70% and the progression-free survival (PFS) was 80–60 and 60%. In all the patients, the copy number variants (CNVs) profile was analyzed using the SNP/CGH array approach. To investigate the clinical relevance of germline *SMARCB1* mutation in AT/RT patients, we performed sequence analysis of the coding regions. The two patients with AT/RT were found to have germline *SMARCB1* mutations. No BRAF mutations were found, and only NTRK gene fusion was present in one patient. We also have examined the association with OS and PFS and different histological subtypes of infant CNS proving that high-grade astrocytoma has better overall survival than other tumor types (*p*: 0.007 and *p*: 0.0590).

**Conclusion:** High-dose chemotherapy regimen represents a valid therapeutic approach for congenital brain tumors with a high rate of response. The molecular analysis has to be analyzed in all infants' brain tumor types. High-grade gliomas are characterized by a better prognosis than other histologies of infant CNS.

## Introduction

Early childhood central nervous system (CNS) neoplasms are rare tumors, and they constitute only 2% of all pediatric brain tumors. The definition of “congenital brain tumor” has been submitted to continuous assessment; several years ago, Jellinger proposed the following: “definitely congenital—symptoms arise at birth or within the first 2 weeks of life; probably congenital—symptoms arise in the first year of life; and possibly congenital—symptoms arise beyond the first year of life” (1). Ellams suggested the following classification: congenital lesion up to 6 weeks from birth, probably congenital to 6 months, and possibly congenital—up to the end of the first year of life (2). The most common congenital neoplasia includes teratomas, astrocytomas (low and high grade), embryonal tumors [medulloblastoma, CNS embryonal tumor not otherwise specified (NOS), atypical teratoid rhabdoid tumors (AT/RT)], choroid plexus tumors, and craniopharyngiomas. Ependimomas and germinomas are less commonly encountered (3, 4). Most of these tumors have a very aggressive behavior, and patients are at a high risk for early mortality after diagnosis. For this reason, few patients are enrolled in clinical trials (5). Particularly, congenital AT/RTs have a fatal prognosis (6). Germline testing for constitutional gene mutations may provide a key information mainly on the AT/RT.

The main prognostic factors that characterize the prognosis of all infant brain tumors, in addition to the type of tumor, could be due to the massive size of these neoplasms at the time of diagnosis, the surgical difficulties in resecting large tumors, and the absence of consolidated therapeutic approaches. As with all brain tumors, surgery is the first fundamental therapeutic approach and the prognosis is highly dependent on the extent of the resection of the tumor. Being that radiotherapy is not recommended for very young patients, intensive chemotherapy regimens with high doses and autologous stem cell transplant (ASCT) after maximal possible surgery could seem to be a helpful adjuvant treatment. Strategically, a multidisciplinary team that includes pediatric neurosurgery and neuro-oncology experts is necessary to approach these complex children.

In this report, we present 10 infants aged less than 12 months with aggressive brain tumors. We evaluated the safety and the effectiveness of high-dose thiotepa and carboplatin/thiotepa followed by stem cell rescue.

## Materials and Methods

### Patient Population

All infants less than 12 months of age with malignant brain tumors admitted between 2003 and 2016 to the Meyer Children's University Hospital of Florence were eligible for this study. Histological diagnosis was examined after admission for adjuvant treatment in all cases by two pathologists. After surgery, tumor specimens were routinely fixed in neutral buffered formol and embedded in paraffin.

### Treatment Protocol

The chemotherapy program was applied to all newly diagnosed patients, aged less than 12 months, at the time of diagnosis. No patient had radiation therapy as first line of treatment.

**Figure 1:**
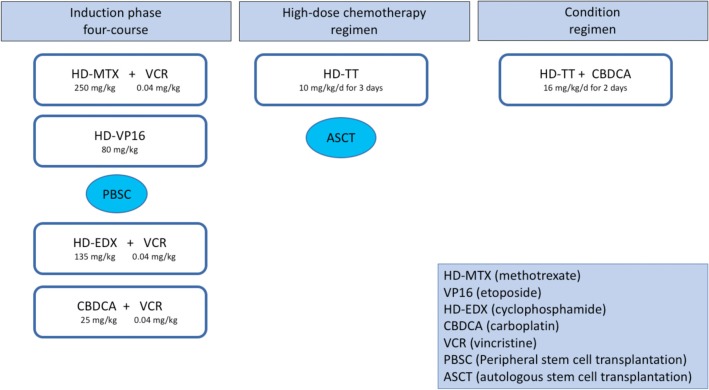
Treatment protocol.

A central line catheter was placed prior to starting standard chemotherapy and high-dose thiotepa and ASCT as previously reported (7). Doses were adjusted for weight. The four-course induction phase included the following: first, methotrexate 250 mg/kg plus vincristine 0.04 mg/kg; second, etoposide 80 mg/kg; third, cyclophosphamide 135 mg/kg plus vincristine 0.04 mg/kg; and finally carboplatin 25 mg/kg as the fourth cycle. Peripheral blood stem cells were collected for rescue therapy after the second course. Intensification and consolidation phases included two high-dose chemotherapy regimens: thiotepa at myeloablative doses (10 mg/kg/day for 3 days) followed by ASCT. The second conditioning regimen also included carboplatin (16 mg/kg/day for 2 days) with thiotepa to improve the response rate (8, 9) (Figure 1).

### Genetic Analysis

Tumor, peripheral blood, and buccal swab DNAs were pulled out using QIAamp Mini Kit (QIAGEN®, Hilden, Germany) according to manufacturers' instructions and quantified by NanoDROP 2000 Spectrophotometer (Thermo Scientific, Waltham, MA, USA).

Sequence analysis of the coding regions of *INI1* gene was prepared with BigDye Terminator v1.1 Cycle Sequencing Kit (Applied Biosystems, Foster City, CA) according to the manufacturer's protocol and sequenced on a 3130 Genetic Analyzer (Applied Biosystems). Primer sequences are available upon request. SNP/CGH array was performed using the Agilent Human Genome CGH Microarray Kit 180K (Agilent Technologies, Santa Clara, CA, USA). Labelling and hybridization were performed following the protocols provided by Agilent, and images of the arrays were acquired with the Agilent C Scanner (Agilent Technologies, Santa Clara, CA, USA) and processed using the Agilent Feature Extraction 10.5 software. The data were analyzed using the Genomic Workbench Standard Edition 5.0 software by the ADM-2 algorithm (breakpoint positions were reported according to Hg19, build 37). Chromosomal analysis was performed on phytohemagglutinin-stimulated peripheral lymphocyte cultures using standard cytogenetic methods (Chromosome Kit P Euro Clone), incubated 72 h at 37°C, and investigated by QFQ-banding analysis. BRAF V600E and NTRK gene fusions were analyzed by immunohistochemistry.

### Statistical Analysis

The main endpoint was the correlation between clinical and molecular factors and overall survival (OS), which included the time from diagnosis to death, whatever the cause. We also evaluated progression-free survival (PFS), which was calculated from the date of diagnosis to the date of relapse or to the date of death. Survival curves (OS and PFS) were estimated using the Kaplan–Meier method with 95% confidence intervals (95% CIs). P values are reported using the log-rank test. The model considered the variables associated with a *P* value < 0.05.

## Results

Ten infants aged less than 12 months with aggressive brain tumors were enrolled in the Neuro-Oncology Unit of Meyer Children's Hospital in Florence. In three cases, the diagnosis was prenatal. Patients with other diagnosis presented the disease from 52 days after birth to 279 days after birth, with a median of 114 days.

Their main clinical and molecular features are summarized in Table 1. Histological diagnoses and tumor grading were carried out based on the 2016 World Health Organization (WHO) criteria (10). The median age at diagnosis was 56 days (range: 1–279 days). Pathological diagnosis was available in all cases: four were AA (WHO-grade III), two GBM (WHO-grade IV), two CNS embryonal tumor NOS (WHO-grade IV), and two AT/RT (WHO-grade IV). The variables considered for each case were as follows: histological type, presence of mutations, localization of primary tumor, and surgery approach.

**Table 1:** Clinical details of infants aged less than 12 months treated for aggressive brain tumors.

**Patient *n***	**Age at diagnosis (days)**	**Histology**	**Resection R0 (gross total resection)** **R2 (partial resection)**	**Genetic analysis**	**Outcome**
1	70	AA	R2	NO	Alive at 87 months
2	1	GBM	R0	NO	Alive at 67 months
3	176	AA	R0	NTRK fusion	Alive at 71 months
4	141	AA	R2	NO	Alive at 56 months
5	1	AA	R2	Somatic trisomy 8 – mosaicism	Alive at 36 months
6	1	AT/RT	R2	Germline SMARCB1/INI1 mutation	DOD at 18 months
7	279	CNS embryonal tumor NOS	R2	NO	Alive at 38 months
8	1	CNS embryonal tumor NOS	R2	NO	Alive at 16 months
9	60	AT/RT	R2	Germline SMARCB1/INI1 mutation	DOD at 6 months
10	52	GBM	R2	NO	DOD at 25 months

*AA, Anaplastic Astrocytoma; GBM, glioblastoma; AT/RT, Atypical Teratoid Rhabdoid Tumor; DOD, Dead of Disease, R0, gross total resection (a resection without visual residual enhancing tumor), R2, partial resection (a resection of only part of the tumor)*.

**Figure 2:**
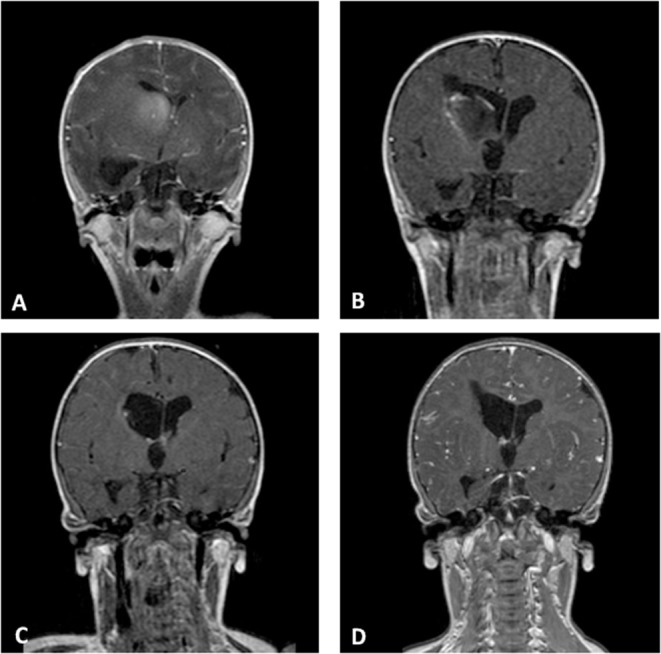
Coronal Gd-enhanced T1-weighted MR scans of a CNS embryonal tumor NOS. **(A)** Preoperative images demonstrating the intraventricular tumor at the right caudate nucleus. **(B)** Postoperative scans after septostomy and biopsy of the lesion. **(C)** Complete response after high-dose chemotherapy and ASCT. **(D)** Last MR follow-up.

In our series, anaplastic astrocytoma was the more frequent histological type with 4 of the 10 cases, all with supratentorial localization. Two other supratentorial tumors were GBM, and the two posterior fossa tumors were AT/RT (in one of these, spinal and cerebrospinal fluid metastases were also present at the diagnosis). Finally, two CNS embryonal tumor NOSs were hemispheric lesions (Figure 2).

**Figure 3:**
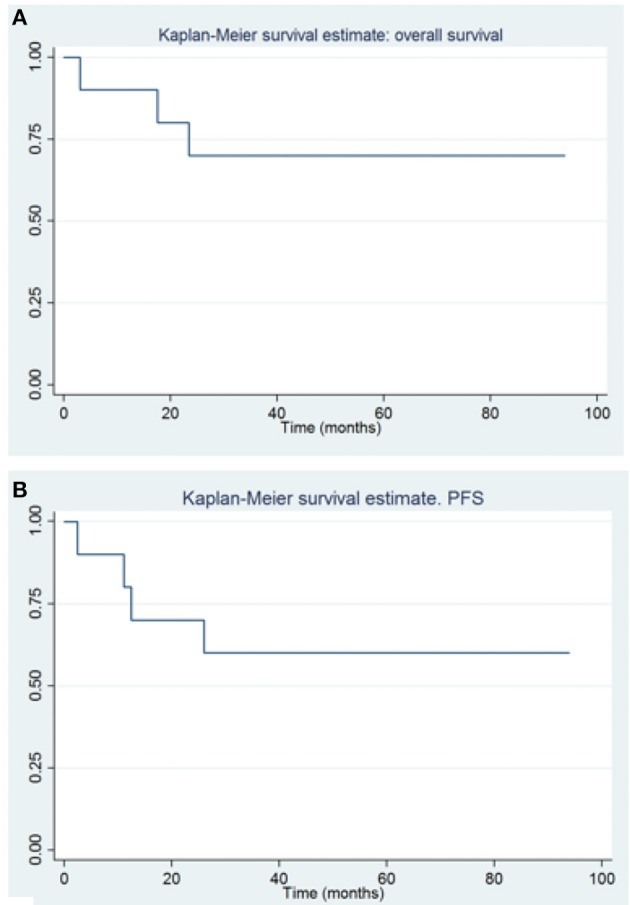
Overall survival **(A)** and progression free survival **(B)** in patients with congenital brain tumors.

The global OS at 1–3–5 years were 90, 70, and 70%, (CI, 47–99, 32–89, and 32–89%, respectively), and the PFS were 80–60 and 60% at 1–3–5 years (CI, 41–95, 25–83, and 25–83%, respectively) (Figure 3). The gold standard treatment for these aggressive tumors is, when possible, maximal surgery (11). In our study, only two patients had GTR. All other patients had only partial resection.

**Figure 4:**
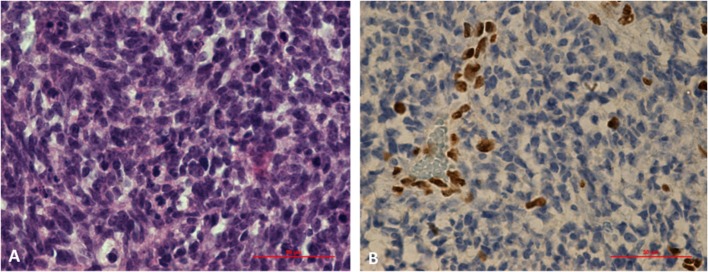
Histological study of an AT/RT with a c.618G>A (p.Trp206*) mutation in exon 5 of *SMARCB1* gene. **(A)** Markedly enlarged atypical epithelioid cells with prominent nucleoli and abundant cytoplasm. Hematoxylin and eosin staining, x60X. **(B)** Results of immunohistochemical staining indicating the loss of SMARCB1 (INI1/hSNF5) expression in neoplastic cells (IHC, ×60).

Germline *SMARCB1* mutations were noted in both patients with AT/RT. One patient with AT/RT had a c.618G>A (p.Trp206^*^) mutation in exon 5 of the *SMARCB1* gene (Figure 4). This variant, already described in rhabdoid tumors, produced a premature stop codon of *SMARCB1* (12). No mutation was identified in the peripheral blood of the father, and unfortunately, the patient was the result of an oocyte donation. The genetic analysis of other patients showed heterozygous c.175C>T mutation in exon 2 of *SMARCB1* in the tumor's DNA.

A genetic rearrangement was found in an AA patient: a duplication of the entire chromosome 8 with a dosage suggestive of genetic mosaics of 15–20% (log2 ratio of +0.3). The supernumerary chromosome 8 was of maternal origin. No mosaicism of supernumerary chromosome 8 was identified in the blood, buccal swab of the patient, and parents' DNA. Chromosome examination on 100 metaphases of the peripheral blood of this patient provided normal results suggesting a plausible somatic trisomy 8 and so excluding a constitutional chromosome 8 mosaicism. No BRAF mutations were found, and only NTRK gene fusion was present in one patient with AA.

**Figure 5:**
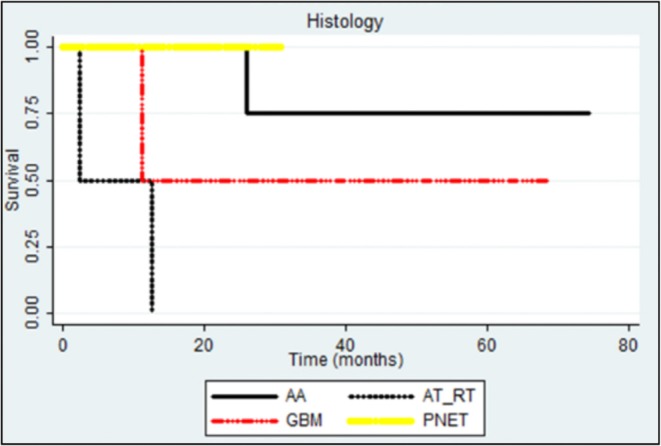
Statistical correlation with OS and PFS with the histology (*p*: 0.007 and *p*: 0.0590).

We also examined the different histologies of infant CNS tumors showing that high-grade gliomas have better prognosis than others; AT/RT has shown a worse prognosis (*p*: 0.007 and *p*: 0.0590; Figure 5).

## Discussion

The incidence of early childhood brain tumors is 1.1–3.6 per 100,000 newborns, and they cause 0.04–0.18% of deaths in infants aged less than 12 months (1, 13).

Malignant congenital brain tumors are rare diseases, and their therapy management is difficult because of the patients' young age. Therefore, there are still no consolidated treatments widely accepted internationally.

The main prognostic factors of CNS tumors are macroscopic disease residual tumor volume after surgery, histology, and presence of metastatic disease. In children younger than 12 months, the main therapeutic approaches are surgery and chemotherapy in order to delay radiotherapy as much as possible. The exposure of immature CNS to radiotherapy can induce early and severe cognitive deficits and severe leukoencephalopathy. Thus, when possible, the best approach for infants remains to be adjuvant chemotherapy after maximal surgery (3, 14).

One-third of all congenital astrocytic tumors are GBM, which mostly grow from the cerebral hemispheres and basal nuclei. These tumors have a high risk of intracranial bleeding and therefore the intralesion hemorrhage may be the first sign of the disease at initial imaging (15). Due to the lack of effective treatments for newborns with malignant astrocytoma, the OS rate remains disheartening. Recently, Guerreiro et al., studying infant gliomas under 1 year of age by genetic analysis, found three subgroups with different outcomes. Group 1 tumors showed ALK/ROS1/NTRK/MET fusions and had a good OS in comparison to older children with HGG. Group 2 hemispheric RAS/MAPK tumors had a very good outcome requiring only a “wait and see” strategy after a safe surgery. Group 3 represented midline LGG characterized by RAS/MAPK alterations. Contrary to what happens in older children, infants with BRAF fused tumors have a dismal outcome. They concluded that an early genetic analysis allows infants with BRAF-fused midline tumors to be included in upfront clinical trials with targeted inhibitors (16).

AT/RT is an extremely aggressive tumor of the CNS, and its biology and histology are similar to the rhabdoid tumor of the kidney, soft tissues, and other sites (17, 18). AT/RT often arises in the posterior fossa, especially in the cerebellum but can grow also in cerebral hemispheres and the brainstem (19).

Germline mutation of the *SMARCB1* gene results in a phenotype known as the “rhabdoid predisposition syndrome,” which increases the risk of developing renal and extrarenal rhabdoid tumors (20).

It is noteworthy that there is a strong correlation between congenital brain tumors and several genetic syndromes (21–25).

The treatment of early childhood brain tumors has always been the subject of wide discussion. The introduction of the prolonged postoperative chemotherapy improved the survival, and it has enabled us to avoid or defer the radiotherapy until relapse.

During the last decades, several therapeutic approaches succeeded obtaining a different survival rate, probably because the same treatment was used for different tumor histologies.

Some studies reported data of congenital brain tumors treated with both chemotherapy and radiotherapy. The 5-year OS in patients subjected to radiotherapy was approximately between 30 and 40%. The morbidity was high irrespective of radiotherapy and most of the patients developed a moderate or severe disability (26, 27).

Di Rocco et al. reported a meta-analysis on 886 children showing minimal side effects in around 50% of patients with congenital brain tumors, whereas more long-term deficits were in infants receiving whole brain irradiation (28).

The “baby brain” study of the Pediatric Oncology Group (POG) analyzed the effect of dose-intensified chemotherapy for infant MB. They utilized cyclophosphamide and vincristine alternating with cisplatin and etoposide. The radiotherapy was done only in patients older than 2 years. Survival utility was not evidenced with this approach compared to other experiences (PFS was 31.8 and OS 39.7% at 5 years). In 1992, they directed the first multicenter trial using adjuvant chemotherapy for children less than 36 months old with malignant brain tumors, deferring the radiotherapy until the age of 3 years. The 5-year OS and PFS rates reached 39.4% and 30%, respectively. The highest proportion of progressive or relapse disease was observed in the first 6 months of chemotherapy (29).

The Children's Cancer Group (CCG) 945 protocol obtained a 3-year PFS and OS of 36 and 50%, respectively, in glioma patients treated with “8 drugs-in-1 day” (vincristine, carmustine, procarbazine, hydroxyurea, cisplatin, cytarabine, dacarbazine, and prednisone) (17).

The CCG with CCG-9921 proposed two more intensive treatment regimes in patients with MB (regimen A: cisplatin, cyclophosphamide, etoposide, and vincristine; regimen B: vincristine, carboplatin, ifosfamide, and etoposide). A 5-year event free-survival (EFS) for regimen A was 38 vs. 26% for regimen B. In patients with CNS embryonal tumor NOS, the rate was low; 5-year OS was 30%. The same results were obtained in AT/RT patients (5-year OS: 29%). In ependymoma patients, 5-year OS was around 58%, and the rate in malignant gliomas was similarly unsatisfactory; the 3-year OS was 42% (30).

The French Society of Pediatric Oncology Baby Brain Protocol (BB-SFOP) adopted the strategy by treating patients with low risk with standard chemotherapy (cycle of carboplatin, procarbazine, etoposide, cisplatin, vincristine, and cyclophosphamide), reserving RT and combined high-dose chemotherapy with ASCT for patients with tumor progression or recurrence. The 3-year OS was 70% for patients with low risk. For patients with high risk, the protocol provided also myeloablative busulfan and thiotepa combining with ASCT and posterior fossa irradiation (TD: 50 Gy). In these patients, neurologic deficits were described, with 5-year OS of 65% in locally relapsed patients (31).

Instead, for children between 2.5 and 3.0 years of age at diagnosis with high-risk tumors, HIT-SKK'87” protocol of the German Society of Pediatric Oncology and Hematology (GPOH) provided the same protocol of patients with low risk. It was expected that after surgery, two cycles of a post-operative induction chemotherapy would be performed. Following the primary treatment, they recommended the maintenance chemotherapy. The radiotherapy was administered at 3 years of age. Radiotherapy was administered immediately in cases of progression or tumor recurrence.

The subsequent HIT-SKK'92 study for children under the age of 3 was aimed at avoiding radiation therapy. The infants were treated with intensive postoperative systemic chemotherapy (cyclophosphamide, methotrexate, vincristine, carboplatin, and etoposide) and intraventricular therapy (2 mg intraventricular methotrexate in single doses via Ommaya reservoir). Craniospinal radiotherapy was done in patients older than 18 months who weren't in remission.

The results obtained for low-risk medulloblastoma were 5-year PFS of 82 ± 9% and OS of 93 ± 6%. The rates obtained for patients with residual disease were 5-year PFS of 50 ± 13% and OS of 56 ± 14%, and for patients with macroscopic metastasis, the outcome was poor with 5-year PFS of 33 ± 14% and OS of 38 ± 15%. Although the study reported a high rate of asymptomatic leukoencephalopathy linked to the intensive use of intrathecal methotrexate, the strategy to postpone craniospinal radiotherapy using postoperative chemotherapy has shown considerable efficacy for controlling tumor growth and survival (32–34).

The role of high-dose, marrow-ablative chemotherapy and ASCT in young patients with MB was investigated by the “Head Start” regimen. In this study, the 5-year OS rate for infant MB was 52% (35). A limitation to this highly toxic approach was the mortality rate of 19%. In Head Start II, high-dose methotrexate was added only in patients with metastatic MB, showing a mortality rate of 5.4% and a 4-year EFS of 51% (36).

Finally, the HIT 2000 trial for MB in children less than 4 years of age considered longer but less dose-intensive induction chemotherapy and a shorter dose-intensive chemotherapy. They planned a tandem high-dose chemotherapy with ASCT for good responders. Radiotherapy was applied to all patients with poor response to induction phase or residual disease after HDCT, whereas it was at the clinician's discretion for patients with residual disease before HDCT. The 5-year EFS and OS rates for the 17 patients were 24 ± 10% and 40 ± 12%, respectively (37, 38).

In 2012, Macy et al. reported a study of five congenital GBM patients who were successfully treated with surgery (one gross total resection, three subtotal resections, and one biopsy only) and a moderately intense chemotherapy regimen (carboplatin and etoposide every 21 days for a range of 6–10 cycles). They obtained good results: four patients were alive in complete remission, showing a disease-free survival range of 30–110 months (median: 36 months). They question the real need for high-dose chemotherapy in light of the obvious clinical progression even in infants treated with aggressive regimens. In their series, they also add that patients with GBM subjected to subtotal resection or biopsy did well, suggesting that aggressive surgery is not necessary because there is a high risk of bleeding causing more morbidity in this fragile population (39).

All our patients were subject to adjuvant chemotherapy after surgery and two cycles consisting of high-dose thiotepa and thiotepa/carboplatin with ASCT, using radiotherapy only in one patient as the second-line treatment. We observed a long-term survival for 5 out of 6 (83%) children. PFS and OS at 5 years were 60 and 70%, respectively. The second-line therapy was used in two patients with GBM. Only one is still alive after radiotherapy treatment at recurrence.

The statistical correlation found with OS and PFS and histology stresses that high-grade astrocytoma has better overall survival than other tumor histology. El-Ayadi et al. have reported a summary of the different studies in infants with primary high-grade gliomas. As confirmed by a previous study (40), they not only added that very young children with AA seem to have better overall survival but also reported important clinicobiological uniqueness of infant AA compared to older patients (41).

Most important complications of chemotherapy are fever with high grade 3–4 neutropenia, moderate and severe anemia, and mucositis. All our patients show delay of growth (> or = 2 SD beyond the mean). The dynamic and evolving aspect of weight and growth is very important; therefore, a careful endocrinological follow-up must be done, considering GH therapy in the future. Treatment for infant brain tumors can reduce the cognitive function. Neurocognitive impairment in survivors is correlated with negative consequents for adulthood, such as unemployment, lower educational achievement, and lower likelihood of marrying. However, it is essential that the tests are submitted to a proper age to assess the actual long-term damage.

In conclusion, congenital brain tumors remain an oncological challenge due to the genetic profile and therapeutic approach. It seems difficult to consolidate the appropriate treatment for malignant congenital brain tumors, given the heterogeneity of histologies. The results extrapolated from international studies show that these complex tumors must be treated by a multidisciplinary neuro-oncology team specialized in the management of newborns/infants in collaboration with pediatric neurosurgery. Given these findings, we believe that future works should focus on multicentric studies to better understand which approach is the most correct.

Despite having a small population, according to our experience, currently HDCT and ASCT represent a valid approach for these very delicate patients.

## Data Availability Statement

The datasets generated for this study are available on request to the corresponding author.

## Ethics Statement

The studies involving human participants were reviewed and approved by Comitato Etico Istituzionale - Meyer Children's Hospital. Written informed consent to participate in this study was provided by the participants' legal guardian/next of kin.

## Author Contributions

MG: literature search, study design, data collection, analysis, interpretation, figures, and writing. LGi: genetic analysis. SD: statistics and data analysis. AB and MS: data collection and pathological analysis. BS and LGe: data collection and writing. IS: idea, data analysis, interpretation, and writing. All authors contributed to manuscript critical revision, read, and approved the submitted version.

### Conflict of Interest

The authors declare that the research was conducted in the absence of any commercial or financial relationships that could be construed as a potential conflict of interest.

## Abbreviations

AA: anaplastic astrocytoma
ASCT: autologous stem cell transplant
AT/RT: atypical teratoid/rhabdoid tumor
CCG: Children's Cancer Group
CNS: central nervous system
GBM: glioblastoma
GTR: gross total resection
HDCT: high-dose chemotherapy
HGG: High grade gliomas
MB: medulloblastoma
OS: Overall survival
PFS: Progression-free survival
POG: Pediatric Oncology Group.
